# My Doctor

**Published:** 1879-10

**Authors:** J. J. Keyes


### MY DOCTOR.

J. J. KEYES.

By the indulgence of the editor, I am going to tell the readers of the BISTOURY some things about my doctor. I may as well state, at the outset, that my doctor is the ideal doctor. I have studied him well, and am satisfied that he is quite near perfection. I take great pride in him; but I am not selfish; I do not wish to monopolize him. I wish all my friends to know him, and when they are sick, to employ him. He is a very rare doctor—is my doctor. I would he were not so rare. I would there were more doctors like him. I would that all the doctors in the world were like him. Perhaps they will be, some day. Let us hope so. In the meantime let us all covet the best doctors, and let all the doctors covet the best gifts.

But what sort of doctor is my doctor? I will tell you.

In the first place, my doctor is a *man*—a true, manly man. I have no objection to the woman doctor; and I should say of her, that she ought to be a true, womanly woman; and that her practice should be mainly among women. But I am discussing doctors generically, and I shall use the word "man" for convenience. Those who chose to read "woman" in place of it, can do so.

Well, then, my doctor is a man—a true, manly man; straight-forward, upright, honorable. He is incapable of meanness. He never sacrifices his manhood. He does not oppress the poor; he does not take advantage of the rich. He does not neglect the poor for fear of small compensation; he does not dance attendance upon the rich, in expectation of large reward.

My doctor is a man whom you can invite to your homes and sick rooms with confidence. Your wives and daughters are as safe with him as they are with you. He is a gentleman, and a true knight of honor, and renders all needed professional attentions to your families, with delicacy and discretion. Being a manly man, he is a man whom you can esteem accordingly. In his personal character and deportment, and in all his transactions, he is a man who commands the respect of the community at large. He is a good citizen as well as a good doctor. He pays his debts, interests himself in public affairs and in the public welfare, and tries to discharge faithfully his obligations as a member of society.

My doctor is not a mischief-making man. He is not a tale bearer, retailing the secrets of one family to another. He is not jealous of his brother doctors—talking against them—decrying them—belittling them, whether they belong to his school, or to some other, with the idea of stealing their practice; nor from any other motive. In short, my doctor is a high minded, honorable, manly man, who stands in his professional boots by reason of his own great merit and sterling worth. He is a true doctor and a true man.

In the second place, my doctor is an educated man. I don't know whether he graduated at Harvard University, or Yale College, or not. I have never thought to ask him. I would not much wonder if he did; but I don't care.

He attended school somewhere, I am sure. At all events, it is evident that he is a man of considerable mental cultivation. He has had sufficient drill in the schools to develop his thinking powers, to sharpen his mental perceptions, and to broaden his intellectual horizon. It was necessary that he should do this; otherwise he might have been as narrow, stupid and mediocre, and as unable to grasp the truths of medical science, as some other doctors I have known; and my doctor would not be content with mediocrity. All the higher professions require in their devotees, in addition to a basis of aptitude and common-sense, a basis of education, of developed faculty, of acquired knowledge in directions other than those of the professions themselves. Otherwise, those who follow them, will, more than likely, be contracted in their views, travel in ruts and circles, and never attain either celebrity or proficiency. My doctor is an educated man. He gave considerable attention to the culture of his mind, as a preparation for those special studies that belong to the realm of science. He then spent a few years in the office of a practicing physician of good repute for medical skill. Of course he attended medical lectures. He also spent a year or two in the great hospitals of New York and Philadelphia. He gave special attention to chemistry, anatomy, and physiology. No man has any business to practice medicine who is ignorant of the structure of the human body, or of the relation of its various parts and organs to each other. He should have a most thorough knowledge of these things, and of the functions of each and all of them.

Suppose a man should try to repair an engine, who knows nothing whatever about machinery. Suppose a man should set up as a tuner of pianos and organs, who knows not how these instruments are constructed, and who cannot name the functions of keys, strings, sounding board, pipe, or pedal. No piece of machinery, no instrument of music, no combination of parts, of any kind, or for any purpose, is so complex, or so difficult of comprehension, as the human body. My doctor understood this fact, years ago, while a student, and spent a great deal of time in acquainting himself with the wonderful, living mechanism which, as a doctor, he knew he would be expected to understand, and which would often be placed in his hands, so to speak, for repairs. No quackery for him! No bungling manipulation of the instrument for him! Although he might fail to reach the ideal of a perfect doctor, he determined that he would never deserve the names, Humbug, Empiric, Charlatan. He resolved to get a "good ready" for his work; and the result was that he became educated in a very high degree and sense.

Furthermore, my doctor is a student, even yet. He did not leave the medical college with the mistaken notion that he "knew it all." At all events, he very soon learned that the study of medicine, though all important, is one thing, and that the practice of it is quite another. Not that the "Books" are so untrustworthy; but because, 1st. The application of knowledge to practical ends, is always more difficult than the acquisition of knowledge ; and because, 2d. New phases and symptoms of disease are continually being developed. By reason of this second fact, a physician must needs be a discerning, studious man. He must not rely wholly upon the "Books, " nor upon the knowledge and experience that he may have previously acquired. If he does, he will often go amiss, and commit serious blunders—fatal blunders, perhaps. My doctor makes a careful study of every serious case that comes under his charge. He keeps his encyclopedia of the practice of medicine and all the most valuable medical journals on his office table, within easy reach. He is familiar with all the latest and best discoveries in chemical and therapeutical science. The healing art is *his business*, and he attends to it, and gives to his patients the benefit of his superior knowledge and skill. And when he has a serious case in hand, he gives it his very best thought and care, never thinking of the "fee," but only of the fact that a precious human life is in danger, and feeling that he must save it if possible. That is the kind of doctor my doctor is; and I have great faith in him; for, not only has he much professional pride; he has, also, a conscientious mind and a humane heart; and he feels it to be incumbent upon him to secure, day by day, the very largest and best preparation for his responsible vocation. "Thoroughly furnished," Saint Paul would say. In a very broad sense, therefore, I consider my doctor an educated man.

It results, of course, in the third place, that my doctor is a skillful diagnostician. (I can't find that word in the dictionaries; but I am sure it ought to be there.) When a man is very sick, a correct diagnosis of his disease is a very important thing. It becomes necessary to know, as nearly as possible, just what ails him. Else, on what principle, or by what rule shall the physician prescribe a remedy. The doctor should be able to judge of a man's disease by the symptons that present themselves. Diagnosis is a word meaning "knowledge through, or by means of." A skillful doctor knows a disease, through, or by means of its symptoms. Accurate diagnosis, therefore, as will readily be seen, requires close, intelligent observation, and careful discrimination. It requires, also, that the doctor be not in too much haste in forming his conclusions, and that he take time, not only to note the symptoms, but also to get from the patient, or from his friends, all the facts bearing upon the case. Lack of caution and judgment in this matter, has undoubtedly, cost many a sick man his life. I once knew a physician who, during my acquaintance with him, never had a case of fever that was not typhoid! All fevers resolved themselves into this one type! and, no matter what a person's ailment might be, calomel was the remedy that he invariably administered. Sometimes his patients got well. Often they died. My doctor is no such doctor. He regards therapeutics as a science, and daily makes it a study. And to succeed well in healing, he understands that he must first ascertain the nature of the disease to be healed. When I apply to him for help, I am morally certain that he will question me and observe my symptoms, until he finds out what is the matter with me, before he writes out a prescription. There are some cases, no doubt, in which the physician must "go it blind" to some extent, and cautiously feel his way along. This whole business of doctoring people is even yet, to some extent, an experimental thing. But far less than formerly. The science of medicine is getting to be understood; and my doctor understands whatever has been, or is now being discovered. He is right up with the times; ahead of the times, in fact; and does'nt propose to allow any new and good thing to elude him. And if you wish to know just what ails you; or if you wish to learn the difference between typhoid and other types of fever; between scarlet rash and small pox; between diphtheria and cankered sore throat—don't go to a quack; don't go to some dear old grandmother who imagines that the spirit of some eminent physician or noted Indian doctor now dead, controls her, and sees and speaks through her; don't go to an old fossil doctor, who has not had a new idea, or made a new discovery during the past forty years. Go to my doctor; a man who is devoted to his profession; a man who is enthusiastic in his pursuit of further knoweledge; a man who practices medicine *con amore;* a man who knows liver complaint from heart disease, and colic from cholera morbus every time.

Time and space both fail me to tell all I know about my doctor. Perhaps he will not thank me for telling what I have already told; for he is a very modest man, and deprecates newspaper and magazine notoriety. But with all due regard to his feelings, I must briefly mention a few things more, before I stop. His name I have solemnly promised not to tell.

There is nothing of sham, or pretence, or brag, or bombast, about my doctor. He does not assume to know more than he does know. He never dresses beyond his means, nor keeps up a style of living that he cannot afford, nor magnifies his professional performances, nor drives through the streets, or along the highways "like Jehu," to convey the impression that he is in great demand, and is doing a "rushing business".

He is not a fussy man, nor a man easily flurried in the sick room. His manner is such as to soothe, not to irritate the patient; and if the case is grave and alarming, he maintains an outward composure.

He is a genial, cheerful man, and has the happy faculty of exciting hopefulness and courage in the minds of those who are placed under his treatment; and, instead of seeking to impress them and their friends, with the fact that they are very "dangerously ill," and that their recovery is very "problematical"—in order that he may add to his laurels, and enhance his reputation by curing them, he rather tries to laugh them out of the notion that they are sick at all,—unless their case is really serious; and even then, he speaks as encouragingly as he dares to speak, knowing that the element of hopefulness is very helpful as a means of cure.

My doctor is a good nurse, also; and will not allow me to die for lack of proper care. If I need more air, light, or sunshine in the room, I have them; if my case requires a certain regimen of clothing, or food, my doctor prescribes it, and insists that I shall have it. If my attendants fall asleep when they ought to keep awake, or if, from any cause, they neglect to administer the medicines at stated intervals, my doctor will not fail to give them a "blessing" at his next visit.

Finally, my doctor is in the habit of curing people. To be sure, everybody must die at one time or another. The time comes when no skill avails, when all remedies fail. But that is no reason why sick people should dismiss their doctors; no reason why doctors should quit their practice; and when a sick man dies, we shall possibly think a very unjust thought, if we think the doctor killed him. Such things, I know, are sometimes said; and when, as sometimes happens, one of *my* doctor's patients dies, I have no doubt that some people are unkind enough to say, or at least, to think, "He killed him." Of course such a remark would not be true. As a rule, I am happy to say, my doctor cures people; and *I* do not believe he *ever* kills them. When they die under his treatment, it is because their time to die has come, and because no medical skill can save them.

Singular that I should feel like using slang in connection with a subject like this; but I do, and I must out with it; my doctor is the "boss" doctor, and I advise all people, everywhere, to employ him when they get sick.

---

To-day has been well called the pupil of yesterday. It is also its descendant and heir.

